# Prolactin serum levels and breast cancer: relationships with risk factors and tumour characteristics among pre- and postmenopausal women in a population-based case–control study from Poland

**DOI:** 10.1038/sj.bjc.6605844

**Published:** 2010-08-24

**Authors:** J M Faupel-Badger, M E Sherman, M Garcia-Closas, M M Gaudet, R T Falk, A Andaya, R M Pfeiffer, X R Yang, J Lissowska, L A Brinton, B Peplonska, B K Vonderhaar, J D Figueroa

**Affiliations:** 1 Cancer Prevention Fellowship Program, Center for Cancer Training, National Cancer Institute, 6120 Executive Blvd (EPS), Suite 150E, MSC 7105, Bethesda, MD 20892, USA; 2 Hormonal and Reproductive Epidemiology Branch, Division of Cancer Epidemiology and Genetics, National Cancer Institute, Bethesda, MD 20892, USA; 3 Department of Epidemiology and Population Health, Albert Einstein College of Medicine, New York, NY 10461, USA; 4 Biostatistics Branch, Division of Cancer Epidemiology and Genetics, National Cancer Institute, Bethesda, MD 20892, USA; 5 Genetic Epidemiology Branch, Division of Cancer Epidemiology and Genetics, National Cancer Institute, Bethesda, MD 20892, USA; 6 Department of Cancer Epidemiology and Prevention, Cancer Center and M. Sklodowska-Curie Institute of Oncology, Warsaw, Poland; 7 Department of Occupational and Environmental Epidemiology, Nofer Institute of Occupational Medicine, Lódź, Poland; 8 Mammary Biology and Tumorigenesis Laboratory, Center for Cancer Research, National Cancer Institute, Bethesda, MD 20892, USA

**Keywords:** lobular carcinoma, molecular subtypes, prolactin, breast cancer

## Abstract

**Background:**

Previous prospective studies have found an association between prolactin (PRL) levels and increased risk of breast cancer. Using data from a population-based breast cancer case–control study conducted in two cities in Poland (2000–2003), we examined the association of PRL levels with breast cancer risk factors among controls and with tumour characteristics among the cases.

**Methods:**

We analysed PRL serum levels among 773 controls without breast cancer matched on age and residence to 776 invasive breast cancer cases with available pretreatment serum. Tumours were centrally reviewed and prepared as tissue microarrays for immunohistochemical analysis. Breast cancer risk factors, assessed by interview, were related to serum PRL levels among controls using analysis of variance. Mean serum PRL levels by tumour characteristics are reported. These associations also were evaluated using polytomous logistic regression.

**Results:**

Prolactin levels were associated with nulliparity in premenopausal (*P*=0.05) but not in postmenopausal women. Associations in postmenopausal women included an inverse association with increasing body mass index (*P*=0.0008) and direct association with use of recent/current hormone therapy (*P*=0.0006). In case-only analyses, higher PRL levels were more strongly associated with lobular compared with ductal carcinoma among postmenopausal women (*P*=0.02). Levels were not different by tumour size, grade, node involvement or oestrogen receptor, progesterone receptor, or human epidermal growth factor receptor 2 status.

**Conclusions:**

Our analysis demonstrates that PRL levels are higher among premenopausal nulliparous as compared with parous women. Among postmenopausal women, levels were higher among hormone users and lower among obese women. These results may have value in understanding the mechanisms underlying several breast cancer risk factor associations.

Prolactin (PRL) is a peptide hormone implicated in growth and differentiation of breast epithelial cells (Das and Vonderhaar, 1997; Maus *et al*, 1999; Vonderhaar, 1999; Clevenger *et al*, 2003). A recent analysis of women from the Nurses’ Health Study (NHS) I and II found that higher PRL levels were associated with breast cancer risk, irrespective of menopausal status (RR=1.3, 95% confidence interval (CI) 1.1–1.6, *P*_het_=0.95) (Tworoger *et al*, 2007a). Previous, smaller case–control and prospective studies (12–71 cases) have generated mixed results regarding the association of PRL levels with breast cancer (Clevenger *et al*, 2003).

Cumulative data have shown parous women to have 15–50% lower PRL levels than nulliparous women, with the majority of this decrease following the first full-term pregnancy (Musey *et al*, 1987; Wang *et al*, 1988; Ingram *et al*, 1990; Eliassen *et al*, 2007). The associations of PRL levels with other known breast cancer risk factors, including: age at menarche and first birth, benign breast disease, and adult body weight, have mostly been null for both pre- and postmenopausal women, even after adjusting for parity (Kwa *et al*, 1976, 1981; Wang *et al*, 1988; Tworoger *et al*, 2006a; Eliassen *et al*, 2007; Su *et al*, 2009).

Conclusions regarding the associations of PRL with breast cancer risk factors and tumour characteristics are largely based on a small number of studies and, in some cases, small populations. Accordingly, further assessment of risk related to PRL levels by patient and tumour characteristics is needed. Using a large population-based case–control study conducted in Poland, we explored the association of PRL levels with known breast cancer risk factors in both pre- and postmenopausal women in our control population. We also examined associations of serum PRL levels with tumour characteristics among the incident cases of breast cancer.

## Materials and methods

Details regarding the population and design of the Polish case–control study have been reported elsewhere (Garcia-Closas *et al*, 2006). Subjects provided written informed consent and the study protocol was approved by ethical boards in Poland and the United States. Briefly, eligible cases were women ages 20–74 years with pathologically confirmed breast cancer living in Warsaw or Lodz, Poland and diagnosed from 2000 to 2003. The Polish Electronic System, a database with demographic information from all residents of Poland, was used to randomly select controls, defined as women without breast cancer, frequency matched to cases on city and age in 5-year categories. In total, 79% of eligible cases (2386) and 69% of eligible controls (2502) consented to participate in an interview regarding breast cancer risk factors. Of these women, 84% of cases and 92% of controls agreeing to the interview provided blood samples. Paraffin-embedded tumour tissue was collected from 87% of cases.

Of 2386 eligible cases with questionnaire data, we selected the subset with invasive cancer tissue prepared as tissue microarrays (TMAs) (*N*=1477). Serum samples were available from 1155 of these women. We excluded women treated before collection of blood (*n*=353) or tumour tissue (*n*=23), resulting in 779 cases for PRL serum analyses. Controls were matched to cases on menopausal status, age (in 5-year increments), time of day of blood draw (within 2 h), study site, and, for premenopausal women, day in menstrual cycle (±2 days).

Prolactin was measured by Quest Diagnostics (San Juan Capistrano, CA, USA) using an immunoassay and concentrations calculated by the ADVIA Centaur instrument (Bayer HealthCare, Tarrytown, NY, USA), which was calibrated with known PRL concentrations. Approximately 3% of samples that included low, medium and high levels of PRL were retested in masked fashion to assess intra- and interbatch variation. The overall coefficient of variation for the PRL serum assay was <5% and the intraclass correlation coefficient was >99%.

Histopathologic features including histology, grade, tumour size, and axillary lymph node metastases were assessed using surgical pathology reports. Tumour features were independently evaluated by the study pathologist (MES). Results for immunohistochemical stains for oestrogen receptor (ER), progesterone receptor (PR), and human epidermal growth factor receptor 2 (HER2) from the invasive cancers, prepared as TMAs with duplicate representation as 0.6-mm diameter cores (Beecher Instruments, Silver Spring, MD, USA), were determined as reported previously (Sherman *et al*, 2007; Yang *et al*, 2007a, 2007b).

### Statistical analysis

Associations between subjects’ demographic and breast cancer risk factors and serum PRL levels were assessed using *T-*test (continuous) and *χ*^2^ analyses (categorical). Analyses were based on natural log transformation of serum PRL levels. The association of PRL concentrations with known risk factors was evaluated among controls using analysis of variance. Details of the entire Poland breast cancer case–control study population with questionnaire data have been reported previously (Garcia-Closas *et al*, 2006).

To determine if serum PRL levels varied by important tumour characteristics, we initially performed polytomous logistic regression to estimate odds ratios (OR) and 95% CI for PRL serum levels with the relevant tumour characteristics as the outcome variables. The final models adjusted for the following variables: time of blood collection in 2-h categories, years of education, age at menarche (⩽12, 13, 14, 15, and ⩾16), age at menopause (<45, 45–49, and ⩾50–54), number of full-term births (0, 1, 2, or 3+), age at first full-term birth (<20, 20–24, 25–29, and ⩾30), family history of breast cancer among first-degree relatives, previous breast disease (history of a benign breast biopsy 1 year before date of diagnosis for cases and date of interview for controls), body mass index (BMI) (<25, 25–29.9, and ⩾30 kg m^–2^), age in 5-year categories, and study site. For premenopausal women, the additional matching variable of menstrual status was also adjusted for in models. For postmenopausal women, models were also adjusted for oral hormone replacement therapy (HRT) use (current/recent use, past use, and ever used oestrogens or combined oestrogen/progestin). Never users of oral HRT were those individuals who used oral HRT for 1 month or less and, among users, current/recent users were those for whom it had been 2 years or less since last use and past users were those for whom the last use was >2 years ago. *P-*values to test for heterogeneity of ORs between tumour characteristics were calculated using logistic regression analyses restricted to cases with the relevant tumour characteristic as the outcome and PRL serum hormone levels as the explanatory variable. Mean serum PRL levels across important tumour characteristics are reported to illustrate the relationship between serum PRL levels and breast cancer histology.

In Supplementary Table 2, we report the associations of breast cancer; with PRL levels, recognising the limitation that blood measurements among patients may reflect disease effects or acute stress responses. For these reasons, this analysis was limited to individuals who had PRL levels within the normal range (i.e., ⩽30 ng ml^–1^), which excluded 4% (*n*=19) of the premenopausal population and 3% (*n*=35) of the postmenopausal from the analysis. PRL levels were significantly different between premenopausal and postmenopausal controls. Therefore, separate quartiles were established for pre- and postmenopausal women using the distributions from the control population. For premenopausal women the quartiles were ⩽7.70, 7.71–10.20, 10.21–15.00, and >15.00 ng ml^–1^. For postmenopausal women the quartiles were ⩽5.30, 5.31–6.70, 6.71–8.40, and >8.40 ng ml^–1^. To estimate the association between PRL serum levels and breast cancer risk, we used conditional logistic regression models separately for pre- and postmenopausal women, as described above for the analysis with tumour characteristics, to compute OR and 95% CIs. Statistical significance was defined as two-sided *P*<0.05 and all analyses were completed with SAS (version 9.0, SAS Institute, Inc., Cary, NC, USA) or STATA (version 9.0, STATA Corporation, College Station, TX, USA) software.

## Results

### Characteristics of study population

The study population consisted of 230 premenopausal and 543 postmenopausal breast cancer cases and an equal number of matched controls. The mean age was approximately 45 (±5.3) years old for premenopausal women and 61 (±7.7) years old for postmenopausal women. With respect to breast cancer risk factors and tumour characteristics, the distributions for the population selected for the analyses presented herein (data not shown) did not significantly differ from the entire Poland breast cancer case–control parent population (Garcia-Closas *et al*, 2006; Yang *et al*, 2007a). Means and frequencies for several breast cancer risk factors are presented in Supplementary Table 1.

### PRL and associations with risk factors among controls in pre- and postmenopausal women

The association of PRL levels with established breast cancer risk factors was examined among controls. Geometric mean PRL levels were 10.9 ng ml^–1^ in premenopausal controls and 7.0 ng ml^–1^ in postmenopausal controls (Table 1). Among premenopausal women, nulliparous women had marginally significantly higher levels of PRL than parous women (mean 13.67 and 10.67 ng ml^–1^, respectively, *P*=0.05) (Table 1). Other comparisons of strata for menstrual and reproductive factors, personal history of benign breast disease and family history were not related to significant differences in PRL levels. Among postmenopausal women, increasing BMI was associated with lower PRL levels (*P*=0.0008). In separate analyses of height and current weight, height was positively associated (*P*=0.04) and current weight was inversely associated (*P*=0.01) with serum PRL levels in postmenopausal women (data not shown), suggesting that the association with current weight is the dominant factor in the inverse association of PRL levels with BMI.

With regard to oral contraceptive use, this population included only 10 current and 28 former premenopausal oral contraceptive users, precluding a meaningful analysis of the relationship of use to PRL levels. Among postmenopausal controls, women currently using HRT had significantly higher PRL levels than never or former users (*P*=0.0006). After mutually adjusting for BMI and HRT, the associations of serum PRL levels with BMI and HRT were still significant (*P*=0.0036 and *P*=0.0028, respectively).

### PRL levels and tumour characteristics in pre- and postmenopausal women

In Supplementary Table 2, we report the association of PRL levels with risk of breast cancer. Among postmenopausal women, higher PRL levels were associated with increased risk of breast cancer (OR=1.76, 95% CI: 1.21–2.57, *P*=0.003) when comparing the highest with the lowest PRL quartile and, among premenopausal women, PRL levels were not associated with increased breast cancer risk.

We further evaluated the association of PRL levels with clinically important tumour characteristics among cases (Table 2). In postmenopausal women, lobular histology was associated with higher levels of PRL than ductal histology (*P*=0.02, Table 2); removing HRT users from the analysis yielded similar conclusions (*P*=0.03). This difference in PRL levels translated into a stronger risk association for lobular (OR=3.04, 95% CI: 1.81–5.91) than ductal (OR=1.62, 95% CI: 1.18–2.23) tumours. No other significant differences in PRL levels were noted across the tumour characteristics of tumour grade, size, node involvement, and ER, PR, or HER2 expression.

## Discussion

Our analysis of serum PRL levels among population-based controls in the Polish study demonstrated significant relationships with three established breast cancer risk factors: nulliparity, among premenopausal women, and HRT and BMI among postmenopausal women. Consistent with previous reports, we found that PRL levels among parous premenopausal women were lower than those among nulliparous women (Musey *et al*, 1987; Eliassen *et al*, 2007). In addition, among premenopausal parous women, levels declined slightly with increasing parity. However, we did not find an association between parity and PRL concentrations among postmenopausal, which contrasts with some reports (Wang *et al*, 1988; Eliassen *et al*, 2007). Lowered PRL levels have been suggested as one of several possible mechanisms that mediate this risk.

In this study, PRL levels were inversely associated with BMI among postmenopausal women, whereas other analyses have shown null (Kwa *et al*, 1976; Tworoger *et al*, 2007b; Su *et al*, 2009) or positive associations (Wang *et al*, 1988; McTiernan *et al*, 2006). Postmenopausal obesity is associated with higher circulating oestrogen levels and increased breast cancer risk in many studies (Key *et al*, 2001). Given that the PRL gene contains an oestrogen response element and that *in vitro* oestrogen upregulates expression of PRL (Duan *et al*, 2008), our inverse association is unexpected. However, in previous analyses from this study, postmenopausal obesity was associated only with larger tumours, rather than breast cancer overall (Garcia-Closas *et al*, 2006), so the subject requires further investigation, ideally by considering distribution of adiposity and concurrent measurements of serum oestrogens.

Although there have been reports demonstrating a positive association of oral contraceptive use with PRL levels (Mishell *et al*, 1977; Scott *et al*, 1978; Clevenger *et al*, 2003), the data regarding HRT are largely null (Castelo-Branco *et al*, 1995; Foth and Romer, 1997; Schlegel *et al*, 1999; Molitch, 2008). In our population, use of both oral contraceptives and HRT were uncommon compared with the United States. Nonetheless, recent/current HRT in the Polish study was significantly associated with higher PRL levels in postmenopausal women. Mutual adjustment of BMI and HRT did not alter these interpretations substantively; both lower BMI and HRT remained related to PRL concentrations. However, these findings need careful interpretation as analyses were based on small numbers of users.

Previous data have suggested that a positive family history of breast cancer may be related to higher PRL levels, especially among premenopausal women (Hankinson *et al*, 1995; Clevenger *et al*, 2003; Eliassen *et al*, 2007). Similarly, we observed increased risk related to increased levels of PRL among women with a family history of breast cancer, but women with a positive family history were relatively uncommon in this data set and results were not statistically significant. Associations of PRL levels with benign breast disease have been mixed and may depend on the particular underlying pathologic condition leading to the development of benign breast disease (Courtillot *et al*, 2005). We did not find an association in Poland, but screening was less common than in some other populations.

In addition, we examined the association of serum PRL levels with tumour characteristics. We did not find significant difference in geometric mean PRL levels by either tumour size or the presence of lymph node metastases, suggesting that PRL levels may not be related to time of clinical diagnosis. In this population, we did identify a stronger relationship between high PRL levels and postmenopausal invasive lobular carcinoma. This finding is interesting and in contrast to previous reports in which no heterogeneity between invasive ductal and lobular cancers was detected (Tworoger *et al*, 2007a). Some previous reports have suggested a relationship between HRT and risk of lobular cancer (Li *et al*, 2008). Our finding of the association of higher PRL levels with invasive lobular carcinoma was independent of HRT use.

Prolactin levels were not related to ER, PR, or HER2 status. These data did not replicate the finding from NHS I and II where the association with PRL was stronger among ER+/PR+ tumours (Tworoger *et al*, 2007a). Our study was truncated at age 74 years and in a largely unscreened population; therefore, the characteristics of our postmenopausal ER+/PR+ cancers may have differed from the NHS. Apart from this analysis, knowledge about relationships of PRL levels and HER2 status are limited and further studies are necessary.

The analyses presented herein have some limitations. Notably, our case–control results must be interpreted with caution as PRL is a stress hormone and we cannot exclude that the relationship with breast cancer was influenced by a stress responses (Freeman *et al*, 2000). In addition, breast tumour cells have been shown to synthesise and secrete PRL in cell culture models (Ginsburg and Vonderhaar, 1995). If PRL levels were affected by tumours or patient stresses, our case–control estimates might be inflated; however, our case–control associations are generally similar to those found in NHS (Tworoger *et al*, 2007a).

In this study, PRL also was measured by an immunoassay that does not discriminate between PRL isoforms, some of which are reported to have varying biological activity (Freeman *et al*, 2000). Regardless, this immunoassay is a widely accepted method for measuring PRL in clinical and epidemiology studies and is currently the only method that can be easily applied to large population-based studies. Our choice of immunoassay also provides the opportunity for our results to be compared with those obtained by others (Hankinson *et al*, 1999; Tworoger *et al*, 2004, 2006b, 2007a). Finally, high mammographic density, which is perhaps the strongest risk factor for non-familial breast cancer apart from age and gender, has been associated with higher PRL levels in some (Boyd *et al*, 2002) but not all (Tamimi *et al*, 2005; Johansson *et al*, 2008) studies. We did not have the ability to examine the association of PRL levels with mammographic density in the current analyses.

The strengths of this study include its population-based design, and extensive collection of risk factor, pathologic and immunohistochemical data. Our analyses were based on incident cases from whom serum was collected at the time of diagnosis of breast cancer. In addition, PRL measurements have been shown to be reliable and most likely a reflection of cumulative exposure over time (Missmer *et al*, 2006; Arslan *et al*, 2008; Tworoger and Hankinson, 2008; Kotsopoulos *et al*, 2010), and hence can be considered a stable marker of exposure and potentially risk. We found that elevated PRL levels were associated with selected breast cancer risk factors and, with the caveats outlined above, also increased breast cancer risk among postmenopausal women. Consistent with previous prospective studies (Tworoger *et al*, 2004) and case–control studies summarised in a recent review (Tworoger and Hankinson, 2008), we found that PRL levels were unrelated to two factors reflecting progression, tumour size, and lymph node metastases. In conclusion, our data suggest that PRL levels may be related to several breast cancer risk factors and could potentially have value in understanding the mechanisms that mediate these factors. Accordingly, continued study of the importance of PRL in breast cancer is warranted.

## Figures and Tables

**Table 1:** Established breast cancer risk factors and PRL serum levels among 773 population controls in the Polish Breast Cancer Study

	**Premenopausal *N*=230**				**Postmenopausal *N*=543**			
	***N***	**Geometric mean PRL (ng ml^–1^)**	**s.d.**	***P-*value**	***N***	**Geometric mean PRL (ng ml^–1^)**	**s.d.**	***P-*value**
Overall geometric mean PRL	230	10.90	1.68		543	7.00	1.74	
								
*Parity*								
Nulliparous	19	13.67	1.58		59	7.19	1.66	
Parous	211	10.67	1.68	0.05^a^	484	6.97	1.61	0.63^a^
								
*Age at menarche*								
⩽12	49	11.57	1.69		118	7.12	1.58	
13	52	11.08	1.74		122	7.51	1.67	
14	76	10.78	1.52		146	6.88	1.54	
15	21	10.67	1.81		57	6.60	1.66	
⩾16	29	9.43	1.84	0.56^b^	94	6.50	1.62	0.19^b^
								
*No. of full-term births*								
Nulliparous	19	13.67	1.58		59	7.19	1.66	
1	73	10.35	1.71		176	7.24	1.69	
2	109	11.09	1.67		225	6.90	1.55	
⩾3	29	9.98	1.58	0.15^b^	83	6.57	1.59	0.43^b^
								
*Age at first full-term birth among parous women*								
⩽20	35	12.04	1.59		144	6.72	1.64	
20–24	107	10.59	1.68		234	7.12	1.59	
25–29	65	11.14	1.68		123	7.10	1.58	
⩾30	23	9.96	1.81	0.3^b^	42	7.23	1.77	0.24^b^
								
*Family history of breast cancer in first-degree relatives*								
No	220	10.86	1.68		506	6.96	1.76	
Yes	10	11.66	1.77	0.67^a^	37	7.45	1.49	0.40^a^
								
*History of benign breast disease*								
No	210	10.86	1.69		500	6.93	1.63	
Yes	17	10.93	1.58	0.78^a^	38	7.66	1.47	0.22^a^
								
*Current BMI (kg m*^*–2*^)								
<25	118	10.99	1.71		165	7.80	1.77	
25–<30	69	10.00	1.67		189	7.13	1.59	
⩾30	42	12.14	1.59	0.15^b^	188	6.22	1.46	0.0008^b^
								
*Age at menopause among postmenopausal women*								
<45					91	7.24	1.65	
45–49					185	6.82	1.60	
⩾50					266	6.37	1.62	0.80^b^
								
*Hormone therapy among postmenopausal*								
Never					422	6.73	1.62	
Current/recent use					31	10.37	1.67	
Past use					29	7.99	1.44	
Ever used oestrogen or progesterone alone					26	7.09	1.44	0.0006^b^

Abbreviations: ANOVA=analysis of variance; BMI=body mass index; PRL=prolactin.

a *P*-values from *t*-test.

b *P-*values from ANOVA.

**Table 2:** Geometric mean PRL serum levels stratified by clinically important tumour characteristics in the Polish Breast Cancer Study

	**Premenopausal**				**Postmenopausal**			
	***N***	**Geometric mean PRL (ng** **ml^–1^)**	**s.d.**	***P-*value**	***N***	**Geometric mean PRL (ng** **ml^–1^)**	**s.d.**	***P-*value**
*Histological type N*=*751*								
Ductal	194	11.02	1.91		425	8.24	1.82	
Lobular	30	12.14	1.44	0.23^a^	102	9.82	2.03	0.02^a^
								
*Tumour size N*=*766*								
⩽2 cm	121	10.98	1.81		280	8.56	1.91	
>2 cm	106	11.33	1.89	0.70^a^	259	8.43	1.82	0.77^a^
								
*Tumour grade N*=*667*								
Well/moderately differentiated	124	10.96	1.86		261	8.75	1.98	
Poorly differentiated	85	11.16	1.86	0.84^a^	201	8.57	1.79	0.73^a^
								
*Axillary node metastasis N*=*755*								
Negative	122	11.20	1.95		335	8.34	1.83	
Positive	103	11.24	1.72	0.97^a^	195	8.66	1.92	0.50^a^
								
*ER N*=*760*								
Negative	83	11.53	1.92		164	8.44	1.64	
Positive	144	10.92	1.80	0.52^a^	369	8.51	1.96	0.87^a^
								
*PR N*=*762*								
Negative	80	10.39	1.88		279	8.51	1.83	
Positive	148	11.60	1.82	0.19^a^	255	8.47	1.91	0.87^a^
								
*ER/PR N*=*759*								
ER+ PR+	121	11.41	1.83		230	8.41	1.95	
ER+PR−	23	8.67	1.56		138	8.73	1.99	
ER−PR+	26	12.38	1.81		25	8.98	7.54	
ER−PR−	57	11.17	1.97	0.19^b^	139	8.35	1.66	0.89^b^
								
*ER/PR/HER2 N*=*752*								
ER+ or PR+ and HER2−	166	11.25	1.81		381	8.59	1.95	
ER+ or PR+ and HER2+	5	8.14	1.71		12	7.52	1.74	
ER− and PR− and HER2+	12	10.14	1.77		38	8.61	1.8	
ER− and PR− and HER2−	45	11.35	2.04	0.64^b^	93	8.28	1.62	0.87^b^

Abbreviations: ANOVA=analysis of variance; ER=oestrogen receptor; HER2=human epidermal growth factor receptor 2; PR=progesterone receptor; PRL=prolactin.

a *P-*values from *t*-test.

b *P-*values from ANOVA.
