# Supplementary material for: Prolactin serum levels and breast cancer: relationships with risk factors and tumour characteristics among pre- and postmenopausal women in a population-based case–control study from Poland
Source: Br J Cancer. 2010 Aug 24;103(7):1097–102. doi: 10.1038/sj.bjc.6605844 (PMC2965860; doi:10.1038/sj.bjc.6605844)
Supplement: Supplementary Tables 1 and 2 [file 6605844x1.doc]

Supplementary Table 1: Characteristics of 776 cases and 773 controls by menopuasal status from the Polish Breast Cancer Study

|  |  | | |  | |  | |  | |  | |  |  | |  |  | |  |  |
| --- | --- | --- | --- | --- | --- | --- | --- | --- | --- | --- | --- | --- | --- | --- | --- | --- | --- | --- | --- |
|  | **Premenopausal** | | | | | | | | | |  | | **Postmenopausal** | | | | | |  |
|  | **Cases N=230** | | | | **Controls N=230** | | | |  | |  | | **Cases *N*=546** | | **Controls *N*=543** | |  | |  |
|  | **Mean** | | **SD** | | **Mean** | | **SD** | | ***P* value** | |  | | **Mean** | **SD** | **Mean** | **SD** | ***P* value** | |  |
| **Age, years** | 45.6 | | 5.2 | | 45.3 | | 5.3 | | 0.49 ^a^ | |  | | 60.7 | 7.9 | 60.7 | 7.7 | 0.99^a^ | |  |
| **Age at menarche, years** | 13.2 | | 1.5 | | 13.7 | | 1.7 | | 0.0006 ^a^ | |  | | 13.7 | 1.7 | 13.7 | 1.8 | 0.66 ^a^ | |  |
| **Age at first birth, years** | 24.1 | | 4.0 | | 24.4 | | 4.3 | | 0.39 ^a^ | |  | | 23.9 | 4.5 | 23.5 | 4.4 | 0.13 ^a^ | |  |
| **Age at menopause,years** | ----------- | | ----------- | | ----------- | | ----------- | | ------------ | |  | | 49.7 | 4.3 | 48.9 | 5.0 | 0.004 ^a^ | |  |
| **Parous, Frequency†** | 202 | | 87.8 | | 211 | | 91.7 | | 0.17^b^ | |  | | 462 | 84.6 | 484 | 89.1 | 0.03^b^ | |  |
| **Family history of breast cancer, Frequency†** | 21 | | 9.1 | | 10 | | 4.4 | | 0.04^b^ | |  | | 54 | 9.9 | 37 | 6.8 | 0.07^b^ | |  |
| **History of benign breast disease, Frequency†** | 24 | | 10.8 | | 14 | | 6.2 | | 0.08^b^ | |  | | 51 | 9.6 | 34 | 6.3 | 0.05^b^ | |  |
| **HRT use, Frequency†** | ----------- | | ----------- | | ----------- | | ------------ | | ----------- | |  | | 166 | 30.7 | 107 | 20.3 | 0.0001^b^ | |  |
|  |  | |  | |  | |  | |  | |  | |  |  |  |  |  | |  |
| † Frequencies represented as N and % instead of Mean and SD | | | | |  | |  | |  | |  | |  |  |  |  |  | |  |
| ^a^ p values from t-test | |  | |  |  | |  | |  | |  | |  |  |  |  |  | |  |
| ^b^p values from χ^2^ test | |  | |  |  | |  | |  | |  | |  |  |  |  |  | |  |

**Supplementary Table 2: Odds ratios and 95%CIs for prolactin serum levels and breast cancer risk in Polish Breast Cancer Study**

|  | **Premenopausal^†^** | | | |  |  |  |  | **Postmenopausal^‡^** | | | |  |  |  |
| --- | --- | --- | --- | --- | --- | --- | --- | --- | --- | --- | --- | --- | --- | --- | --- |
|  | **Cases** |  | **Controls** |  |  |  |  |  | **Cases** |  | **Controls** |  |  |  |  |
| **Prolactin** | **N=217** | **%** | **N=223** | **%** | **OR** | **95% CI** | |  | **N=518** | **%** | **N=536** | **%** | **OR** | **95% CI** | |
| **Quartile 1** | 55 | 25 | 57 | 26 | 1.00 |  |  |  | 107 | 21 | 143 | 27 | 1.00 |  |  |
| **Quartile 2** | 59 | 27 | 56 | 25 | 0.92 | 0.50 | 1.68 |  | 105 | 20 | 133 | 25 | 0.93 | 0.64 | 1.37 |
| **Quartile 3** | 48 | 22 | 55 | 25 | 0.79 | 0.41 | 1.49 |  | 119 | 23 | 126 | 24 | 1.21 | 0.81 | 1.81 |
| **Quartile 4** | 55 | 25 | 55 | 25 | 0.74 | 0.39 | 1.41 |  | 187 | 36 | 134 | 25 | 1.76 | 1.21 | 2.57 |
|  |  |  |  |  |  |  |  |  |  |  |  |  |  |  |  |

**^†^**Adjusting for BMI, education, family history of breast cancer among first-degree relatives, previous breast disease, age at menarche, age at first full-term birth, and number of full-term births.

**^‡^**Adjusting for BMI, education, family history of breast cancer among first-degree relatives, previous breast disease, oral HRT use, age at menarche, age at menopause, age at first full-term birth, and number of full-term births.
